# Critical Prognostic and Predictive Factors in Colorectal Liver Metastasis: A Thorough Analysis of Existing Literature and Future Outlook

**DOI:** 10.3390/jcm15134907

**Published:** 2026-06-24

**Authors:** Paul Pasca, Flaviu Ionut Faur, Cosmin Burta, Dan Brebu, Carmen Neamtu, Vlad Braicu, Ciprian Duta, Ioana Adelina Faur, Razvan Danau, Amadeus Dobrescu, Marius Murariu

**Affiliations:** 1 IInd Surgery Clinic, Timisoara Emergency County Hospital, 300723 Timisoara, Romania; paul.pasca@umft.ro (P.P.); mihai.burta@umft.ro (C.B.); brebu.dan@umft.ro (D.B.); braicu.vlad@umft.ro (V.B.); duta.ciprian@umft.ro (C.D.); adelina.clim@umft.ro (I.A.F.); dobrescu.amadeus@umft.ro (A.D.); 2 X Department of General Surgery, “Victor Babes” University of Medicine and Pharmacy Timisoara, 300041 Timisoara, Romania; 3 Doctoral School of Medicine, “Victor Babes” University of Medicine and Pharmacy Timisoara, Eftimie Murgu Square 2, 300041 Timisoara, Romania; 4 Multidisciplinary Doctoral School, “Vasile Goldiș” Western University of Arad, 310025 Arad, Romania; 5 Ist Clinic of General Surgery, Arad County Emergency Clinical Hospital, 310158 Arad, Romania; neamtu.carmen@uvvg.ro; 6 Department of General Surgery, Faculty of Medicine, “Vasile Goldiș” Western University of Arad, 310025 Arad, Romania; 7 Carol Davila University of Medicine and Pharmacy, 020021 Bucharest, Romania; 8 “Prof. Dr. Th. Burghele” Clinical Hospital, Urology Department, Carol Davila University of Medicine and Pharmacy, 050659 Bucharest, Romania; 9 Abdominal Surgery and Phlebology Research Center, “Victor Babes” University of Medicine and Pharmacy Timisoara, 300041 Timisoara, Romania; murariu.marius@umft.ro; 10 First Surgery Clinic, “Pius Brinzeu” Clinical Emergency Hospital, 300723 Timisoara, Romania

**Keywords:** microsatellite instability (MSI), *BRAF* mutation, *KRAS* mutation, *NRAS* mutation, tumor budding, inflammatory markers, desmoplastic growth pattern (dHGP)

## Abstract

**Background:** Colorectal cancer (CRC) prognosis, particularly in liver metastasis (CRLM), is influenced by histopathological and molecular factors. **Methods:** A narrative analysis of the specialized literature was conducted using databases such as PubMed, MEDLINE, Scopus, and Embase. The review focused on original articles published between 2005 and 2025. **Results:** Lymph node involvement is a critical prognostic factor, with lymph node-positive CRC correlating with increased risk of liver metastasis and significantly reduced survival rates. Poorly differentiated tumors (G3) exhibit a higher likelihood of metastasis, including liver involvement, and are associated with worse clinical outcomes. Vascular emboli and perineural invasion are indicative of hematogenous spread and higher metastatic potential, leading to poorer survival outcomes. Genetic mutations, such as *KRAS*, *NRAS*, and *BRAF*, are associated with therapy resistance, complicating treatment and highlighting the importance of personalized approaches. MSI-H and HER2 amplification further affect treatment response, with MSI-H tumors showing a favorable response to immunotherapy, while HER2-positive CRCs may benefit from targeted therapies. Tumor budding, high levels of which predict poor survival, is another key histopathological feature associated with aggressive metastatic behavior. Systemic inflammatory markers, such as the Neutrophil-to-Lymphocyte Ratio (NLR), Platelet-to-Lymphocyte Ratio (PLR), and C-Reactive Protein-to-Albumin Ratio (CAR), offer prognostic insights into CRLM patient survival. **Conclusions:** Histopathological features, molecular alterations, and immune microenvironment factors significantly impact the prognosis of CRC with liver metastasis. The integration of molecular profiling, immunotherapy, and targeted therapies offers promise for improving treatment outcomes. Personalized treatment strategies, incorporating these factors, are essential for overcoming therapy resistance and improving survival in CRLM patients.

## 1. Introduction

Colorectal cancer is one of the most common malignancies worldwide and remains a leading cause of cancer-related death [1]. The liver is the most frequent site of distant metastasis in CRC, and the presence, extent, and biological behavior of CRLM strongly influence treatment selection and survival [2,3].

**Figure 1:**
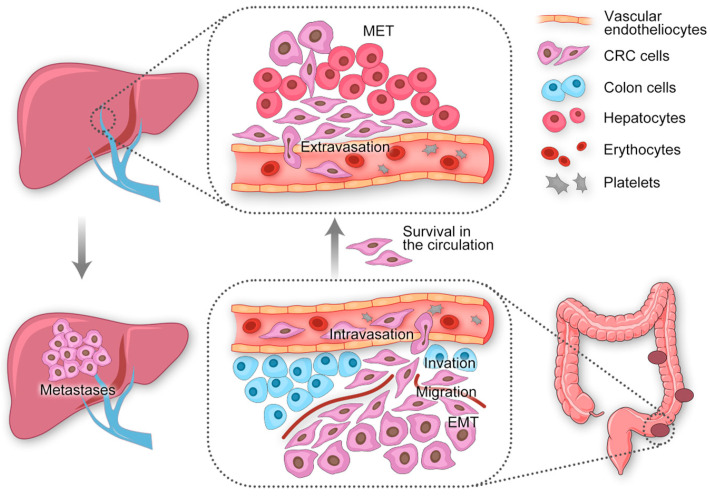
Integrated prognostic framework in colorectal cancer liver metastasis (CRLM). The figure illustrates the interaction between histopathological factors (lymph node involvement, tumor differentiation, vascular invasion, tumor budding, and histopathological growth patterns), molecular alterations (*KRAS*, *NRAS*, *BRAF*, MSI/dMMR, HER2, POLE/POLD1), immune microenvironment components (tumor-infiltrating lymphocytes, macrophages, immune checkpoints), and systemic inflammatory markers (NLR, PLR, CAR, SII) contributing to metastatic progression, treatment response, and survival outcomes.

Although surgical resection may provide long-term survival in selected patients, CRLM management increasingly depends on multidisciplinary integration of systemic therapy, molecular profiling, pathological risk assessment, and patient-specific disease biology [2,3,4]. Classical prognostic models, including clinical scores based on nodal status, disease-free interval, tumor burden, CEA level, and metastatic size, remain clinically useful but do not fully capture tumor heterogeneity or therapeutic response [4,5]. (Figure 1, Table 1).

Histopathological features such as lymph node involvement, tumor differentiation, vascular invasion, perineural invasion, tumor budding, and histopathological growth patterns provide essential information on metastatic potential and recurrence risk [4,5,6,7,8,9]. Molecular factors, including *KRAS*, *NRAS*, *BRAF*, MSI/dMMR status, HER2 amplification, and POLE/POLD1 alterations, influence both prognosis and treatment selection in metastatic CRC [2,3,10,11,12,13,14,15,16].

The tumor immune microenvironment has become increasingly relevant because immune cell density, spatial distribution, and inflammatory contexture influence colorectal cancer progression and treatment response [6,17,18]. Systemic inflammatory markers, including NLR, PLR, CAR, and SII, offer inexpensive and easily measurable prognostic information, although cut-off values and clinical implementation remain heterogeneous [19,20,21,22,23].

Transcriptomic and cDNA-based studies further demonstrate that metastatic progression is not determined by a single pathway but by coordinated programs involving epithelial–mesenchymal transition, stromal activation, immune evasion, angiogenesis, and treatment resistance [24,25,26,27,28]. These emerging molecular frameworks support the transition from conventional clinicopathological assessment toward multidimensional prognostic models [24,25,26,27,28,29].

The aim of this review is to provide a structured synthesis of histopathological, molecular, inflammatory, and immune microenvironment factors relevant to CRLM prognosis and treatment selection [2,3,6,7,8,9,17,18]. The review distinguishes predictive factors related to the biological propensity for liver metastasis from prognostic factors associated with survival after metastatic disease has occurred [4,5,6,7,8,9]. Unlike previous reviews focused predominantly on surgical strategies or isolated biomarkers, the present review integrates these complementary determinants into a unified biomarker-driven framework for CRLM risk stratification [6,7,8,9,17,18,24,25,26,27,28,29].

**Table 1:** Overview of prognostic and predictive factors in colorectal cancer liver metastasis.

Category	Factor	Clinical Relevance
Histopathological	Lymph node involvement	Predictor of metastatic risk and survival [4,5]
Histopathological	Tumor differentiation	Indicator of biological aggressiveness [4,5]
Histopathological	Vascular/perineural invasion	Marker of invasive and metastatic behavior [4,5]
Histopathological	Tumor budding	Adverse prognostic feature and invasion marker [9]
Histopathological	Histopathological growth pattern	Prognostic pattern in liver metastases [8]
Molecular	*KRAS*/*NRAS* mutations	Anti-EGFR resistance and adverse biology [10,11,12]
Molecular	*BRAF* V600E mutation	Poor prognosis and targetable alteration [13]
Molecular	MSI-H/dMMR	Predictive marker for immune checkpoint inhibition [14,15,16]
Molecular	HER2 amplification	Targetable alteration in selected RAS wild-type disease [29,30,31,32]
Immune/inflammatory	TILs, Immunoscore, NLR, PLR, CAR, SII	Immune and systemic inflammatory prognostic markers [6,17,18,19,20,21,22,23]

## 2. Materials and Methods

This narrative review synthesizes current evidence regarding histopathological, molecular, inflammatory, and immunological determinants of CRLM using a structured evaluation of the available literature [2,3]. The analysis was designed to clarify how established and emerging biomarkers contribute to prognosis, metastatic progression, and treatment selection in metastatic colorectal cancer [2,3,10,11,12,13,14,15,16].

The literature search was performed in PubMed, MEDLINE, Scopus, and Embase using combinations of the following terms: colorectal liver metastasis, CRLM, *KRAS*, *NRAS*, *BRAF*, MSI-H, dMMR, HER2 amplification, POLE, POLD1, PD-L1, tumor budding, histopathological growth pattern, immune microenvironment, Immunoscore, neutrophil-to-lymphocyte ratio, platelet-to-lymphocyte ratio, C-reactive protein-to-albumin ratio, systemic immune-inflammation index, transcriptomic profiling, and cDNA profiling [2,3,8,9,10,11,12,13,14,15,16,19,20,21,22,23,24,25,26,27,28,29].

Eligible studies included clinical trials, cohort studies, systematic reviews, meta-analyses, consensus statements, and guideline-based publications addressing CRC, CRLM, prognostic biomarkers, predictive biomarkers, or metastatic treatment selection [2,3]. Studies were prioritized when they provided clinically relevant data on human CRC or CRLM populations, biomarker assessment, treatment response, or survival outcomes [2,3,10,11,12,13,14,15,16,19,20,21,22,23].

Studies not directly related to colorectal cancer, liver metastasis, biomarker-driven prognosis, or metastatic treatment were excluded [2,3]. Preclinical studies were considered only when they supported clinically relevant translational concepts, such as stromal signatures, transcriptomic subtypes, or immune microenvironment mechanisms [24,25,26,27,28].

Relevant information from the selected literature was qualitatively synthesized with emphasis on clinically relevant histopathological, molecular, inflammatory, immune, and transcriptomic biomarkers associated with CRLM prognosis and treatment selection. Numerical claims lacking consistent validation across the literature were removed or reformulated to avoid unsupported overstatement. For conceptual clarity, the reviewed literature was analyzed according to two major clinical settings: (1) predictive factors associated with the development of colorectal liver metastasis in patients with initially non-metastatic colorectal cancer, and (2) prognostic factors influencing survival and treatment outcomes in patients with established CRLM.

## 3. Results

The selected literature supports a multidimensional approach to CRLM prognosis, in which clinicopathological variables remain important but are increasingly complemented by molecular, inflammatory, immune, and transcriptomic biomarkers [2,3,4,5,6,7,8,9,17,18,19,20,21,22,23,24,25,26,27,28,29].

### 3.1. Lymph Node Involvement, Tumor Differentiation, and Metastatic Potential

Lymph node involvement remains one of the most established adverse prognostic factors in colorectal cancer and is strongly associated with increased risk of distant metastasis and inferior survival [4,5]. In patients who develop CRLM, nodal positivity of the primary tumor reflects aggressive tumor biology and supports closer surveillance and systemic treatment consideration [2,3,4,5].

Tumor differentiation provides additional information regarding tumor aggressiveness, because poorly differentiated tumors tend to demonstrate increased invasive capacity, genomic instability, and metastatic potential [4,5]. Although differentiation alone is insufficient for therapeutic decision-making, it remains an important component of routine pathology reporting and risk assessment [2,3,4,5].

Vascular invasion and perineural invasion are markers of local aggressiveness and dissemination capacity, and their presence is associated with increased recurrence risk and adverse oncological outcomes [4,5]. These features should be interpreted alongside nodal status, tumor burden, and molecular markers rather than as isolated determinants of prognosis [2,3,4,5,10,11,12,13,14,15,16].

### 3.2. Histopathological Growth Patterns in CRLM

Histopathological growth patterns describe the interface between metastatic tumor tissue and adjacent liver parenchyma and include desmoplastic, pushing, replacement, and less common mixed patterns [8]. International consensus guidance has standardized HGP scoring and supports its use as a reproducible pathological descriptor in liver metastases [8] (Table 2).

The desmoplastic growth pattern is generally associated with improved prognosis, stronger stromal reaction, and more organized immune infiltration [8,17]. In contrast, replacement-type growth is associated with vessel co-option, reduced therapeutic vulnerability to anti-angiogenic approaches, and inferior outcomes in several CRLM cohorts [8,18].

HGP assessment remains incompletely implemented in routine clinical workflows, but it may complement classical staging and molecular profiling in future risk-stratification models [8,18]. For this reason, HGP should be regarded as an emerging biomarker with strong biological plausibility but variable standardization across institutions [8,18].

### 3.3. Tumor Budding and Immunohistochemical Markers

Tumor budding refers to the presence of isolated tumor cells or small clusters composed of up to four tumor cells located at the invasive front of colorectal carcinomas and is considered a histopathological surrogate of epithelial–mesenchymal transition (EMT), tumor invasiveness, and metastatic potential [9]. EMT is characterized by the loss of epithelial polarity and adhesion molecules, particularly E-cadherin, together with increased migratory and invasive capabilities of tumor cells [9]. The International Tumor Budding Consensus Conference (ITBCC) established standardized recommendations for tumor budding assessment based on hotspot analysis and bud quantification, significantly improving interobserver reproducibility and clinical applicability in colorectal cancer pathology reporting [9].

High-grade tumor budding has consistently been associated with adverse oncological features, including lymph node metastasis, vascular invasion, distant dissemination, local recurrence, and reduced survival [9]. Multiple studies demonstrated that increased tumor budding correlates with biologically aggressive disease and may identify patients at higher risk for metastatic progression even in earlier disease stages [9]. In the setting of colorectal liver metastasis (CRLM), tumor budding should not be interpreted as an isolated histological feature but rather integrated into a broader pathological and molecular framework, including histopathological growth patterns (HGP), tumor differentiation, lymphovascular invasion, perineural invasion, microsatellite instability, and molecular alterations such as *KRAS*, *NRAS*, and *BRAF* mutations [8,9,10,11,12,13,14,15,16]. Emerging evidence also suggests that tumor budding may correlate with resistance to systemic therapy and increased postoperative recurrence risk following hepatic resection [8,9].

Immunohistochemical (IHC) biomarkers provide additional clinically relevant information regarding tumor lineage, differentiation, molecular subtype, immune microenvironment, and therapeutic vulnerability [6,11,14,15,16,17,29,30,31,32]. Among these, CDX2 expression represents an important marker of intestinal differentiation, and loss of CDX2 has been associated with aggressive tumor biology, metastatic dissemination, chemotherapy resistance, and poorer survival outcomes in colorectal cancer [29,30]. Abnormal p53 expression, frequently reflecting underlying *TP53* alterations, has also been linked to genomic instability, tumor progression, and inferior prognosis in metastatic colorectal cancer [30].

HER2 overexpression or amplification identifies a distinct molecular subgroup of metastatic colorectal cancer, particularly among *RAS* wild-type tumors, and may predict responsiveness to HER2-targeted therapies, including trastuzumab-based combinations [14,15,16,31]. Similarly, mismatch repair (MMR) protein assessment by IHC is now routinely integrated into clinical practice because deficient mismatch repair (dMMR) and microsatellite instability-high (MSI-H) tumors demonstrate distinct biological behavior and increased sensitivity to immune checkpoint inhibition [2,3,14,15,16]. In contrast, PD-L1 expression alone has shown inconsistent prognostic and predictive value in colorectal cancer and currently lacks the clinical maturity observed in other malignancies such as non-small cell lung cancer or melanoma [14,15,16,32].

Beyond tumor-cell biomarkers, immunohistochemistry also enables characterization of the immune microenvironment through evaluation of tumor-infiltrating lymphocytes (TILs), macrophage polarization, stromal activation, and immune checkpoint expression [6,14,15,16,17]. Markers such as CD3, CD8, CD68, and CD163 provide insight into immune-cell composition and may help identify immunologically “hot” versus immunosuppressive tumors [6,10]. However, despite promising translational data, many stromal and immune-related IHC biomarkers remain exploratory and are not yet standardized for routine clinical use [29,30,31,32].

### 3.4. Immune Landscape and Metastatic Niche

**Figure 2:**
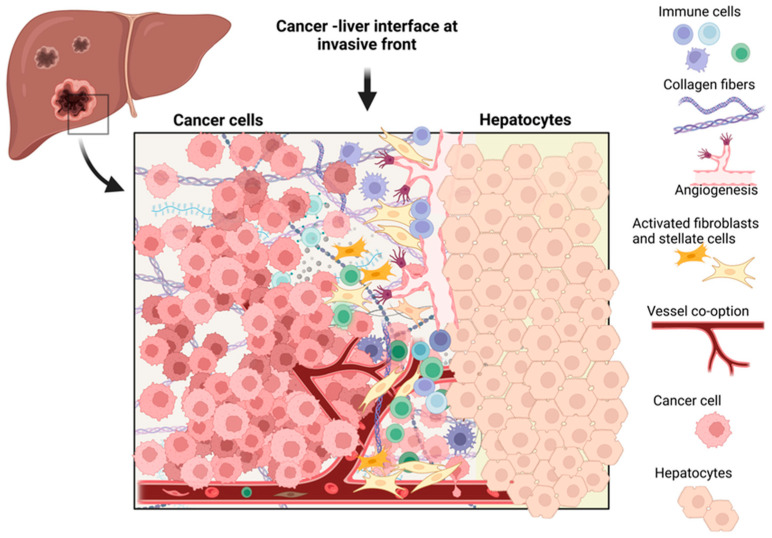
Immune and stromal microenvironment of colorectal cancer liver metastasis. The figure illustrates interactions between metastatic tumor cells, cancer-associated fibroblasts, tumor-associated macrophages, Kupffer cells, dendritic cells, endothelial cells, and cytotoxic T lymphocytes within the hepatic metastatic niche. Immune checkpoint pathways, including PD-1/PD-L1 contribute to immune evasion and metastatic progression.

The immune contexture of colorectal cancer—including the density, composition, functional status, and spatial distribution of immune cells within the tumor microenvironment—has major prognostic significance and directly influences metastatic behavior and treatment response [6]. High densities of CD3+ and CD8+ cytotoxic T lymphocytes at both the tumor core and invasive margin have consistently been associated with improved disease-free and overall survival, forming the biological foundation of the Immunoscore concept [6,17]. Several studies demonstrated that immune-rich tumors exhibit lower metastatic potential and improved responsiveness to systemic therapies, including immunotherapy [6,17] (Figure 2).

The metastatic liver niche differs substantially from the immune microenvironment of the primary colorectal tumor because it is shaped by the liver’s intrinsic tolerogenic physiology [7,18]. The hepatic microenvironment contains sinusoidal endothelial cells, Kupffer cells, stellate cells, dendritic cells, stromal fibroblasts, regulatory T cells, and tumor-associated macrophages, all of which contribute to immune modulation and maintenance of local immune tolerance [7,18]. Continuous exposure of the liver to gut-derived antigens promotes an immunosuppressive baseline state that metastatic tumor cells may exploit to evade immune surveillance and establish metastatic colonization [7,18].

This hepatic immune tolerance may partially explain why liver metastases sometimes demonstrate reduced responsiveness to immune checkpoint inhibitors compared with extrahepatic metastatic sites, even in tumors harboring potentially immunogenic molecular profiles such as MSI-H or high tumor mutational burden [7,14,15,16,18]. Immune exclusion, impaired T-cell infiltration, macrophage-mediated suppression, and stromal remodeling all contribute to the creation of an immunosuppressive metastatic niche [7,18].

Tumor-associated macrophages (TAMs), particularly CD68+/CD163+ polarized macrophages, have been associated with poorer survival outcomes, enhanced angiogenesis, extracellular matrix remodeling, and facilitation of metastatic progression [7,18,24,25,26,27,28]. Cancer-associated fibroblasts (CAFs) also contribute significantly to metastatic niche formation by producing extracellular matrix components, cytokines, chemokines, and growth factors that promote tumor-cell survival and immune evasion [24,25,26,27,28]. In parallel, immune checkpoint pathways involving PD-1/PD-L1 interactions suppress cytotoxic T-cell activity and further facilitate immune escape [14,15,16].

Recent translational studies suggest that integrating molecular profiling with immune microenvironment characterization may improve prognostic stratification and help identify patients more likely to benefit from immunotherapy, targeted therapies, or combination treatment strategies [7,18,24,25,26,27,28,29]. The increasing recognition of immune heterogeneity in CRLM supports the development of future biomarker-driven therapeutic algorithms combining histopathological, molecular, and immune parameters in order to optimize personalized treatment approaches.

### 3.5. Inflammatory Markers and Their Prognostic Value

Systemic inflammation plays a central role in colorectal cancer progression and metastatic dissemination by promoting angiogenesis, tumor-cell proliferation, immune escape, endothelial activation, and protection of circulating tumor cells from immune-mediated destruction [19,20,21,22,23]. In colorectal cancer liver metastasis (CRLM), inflammatory biomarkers derived from routine peripheral blood tests have gained increasing clinical relevance because they are inexpensive, reproducible, widely available, and easily integrated into preoperative risk stratification models [19,20,21,22,23]. Elevated inflammatory indices have consistently been associated with inferior oncological outcomes, including reduced overall survival (OS), decreased recurrence-free survival (RFS), increased postoperative recurrence risk, and more aggressive metastatic behavior [19,20,21,22,23] (Table 3).

The neutrophil-to-lymphocyte ratio (NLR) reflects the balance between systemic inflammatory activation and anti-tumor immune surveillance mediated by lymphocytes [19,20]. Neutrophils contribute to tumor progression through the secretion of proangiogenic and proinflammatory mediators, including vascular endothelial growth factor (VEGF), interleukin-6, and matrix metalloproteinases, whereas lymphocytes are essential for cytotoxic anti-tumor immune responses [19,20]. Elevated NLR has been consistently associated with poorer prognosis in patients with CRLM, particularly in those undergoing liver resection or systemic chemotherapy [19,20]. In a meta-analysis evaluating inflammatory biomarkers in CRLM, elevated preoperative NLR was significantly associated with reduced overall survival and recurrence-free survival after hepatic resection [19]. Similarly, Kishi et al. demonstrated that patients with elevated NLR had significantly inferior survival outcomes following systemic chemotherapy for colorectal liver metastases, supporting the role of NLR as a marker of biologically aggressive disease and impaired anti-tumor immunity [20]. Reported cut-off values vary substantially between studies, most commonly ranging between 3 and 5, which limits universal standardization and broad clinical implementation [19,20].

The platelet-to-lymphocyte ratio (PLR) represents another clinically relevant inflammatory biomarker reflecting the interaction between platelet-mediated tumor promotion and lymphocyte-dependent immune surveillance [21]. Platelets contribute to metastatic progression by facilitating tumor-cell adhesion to vascular endothelium, protecting circulating tumor cells from immune elimination, and promoting angiogenesis through the release of growth factors such as VEGF and platelet-derived growth factor (PDGF) [21]. Elevated PLR has been associated with adverse survival outcomes in metastatic colorectal cancer and particularly in CRLM patients undergoing hepatectomy [21]. Neofytou et al. reported that elevated PLR was significantly associated with poorer prognosis following liver resection for colorectal liver metastases, supporting its potential role as a complementary inflammatory biomarker alongside NLR [21]. However, substantial heterogeneity exists regarding PLR thresholds, with most studies using cut-off values ranging from 150 to 300, further emphasizing the need for prospective validation and standardization before routine clinical adoption [21].

The C-reactive protein-to-albumin ratio (CAR) integrates two clinically important parameters: systemic inflammation and nutritional status [22]. C-reactive protein (CRP) is an acute-phase reactant synthesized in response to inflammatory cytokines, whereas albumin reflects both nutritional reserve and chronic systemic inflammatory burden [22]. Elevated CAR has been associated with impaired postoperative recovery, decreased tolerance to systemic chemotherapy, increased recurrence risk, and reduced survival in patients with metastatic colorectal cancer [22]. Shibutani et al. demonstrated that elevated CAR was an independent adverse prognostic factor in metastatic colorectal cancer patients treated with chemotherapy, indicating that combined inflammatory and nutritional impairment contributes significantly to unfavorable oncological outcomes [22]. Nevertheless, CAR interpretation remains complicated by confounding factors including infection, hepatic dysfunction, cachexia, and treatment-related inflammatory changes [22].

The systemic immune-inflammation index (SII), calculated using neutrophil, platelet, and lymphocyte counts, has emerged as a more comprehensive inflammatory biomarker integrating multiple components of tumor-associated inflammation and host immune response [22,23]. Elevated SII reflects increased systemic inflammatory activation combined with impaired adaptive anti-tumor immunity and has been associated with more aggressive disease biology, higher recurrence rates, and inferior survival outcomes in CRLM patients [22,23]. Deng et al. reported that elevated SII was significantly associated with poorer prognosis in patients with colorectal liver metastases, reinforcing its potential role in inflammatory risk stratification and prognostic modeling [23]. However, despite promising results, SII remains insufficiently standardized because reported thresholds vary considerably between studies and are influenced by treatment setting, systemic therapy exposure, and patient selection criteria [22,23]. Consequently, although inflammatory biomarkers including NLR, PLR, CAR, and SII provide clinically relevant prognostic information, their integration into routine CRLM management still requires prospective multicenter validation and harmonization of clinically applicable cut-off values [19,20,21,22,23].

### 3.6. Molecular and Histopathological Characteristics as Prognostic and Predictive Factors

Molecular profiling has become a fundamental component of modern metastatic colorectal cancer management because it provides biomarkers with both prognostic and predictive significance, directly influencing therapeutic selection and survival outcomes [2,3]. Current guideline-based management recommends routine assessment of *KRAS*, *NRAS*, *BRAF V600E*, and MSI/dMMR status in all patients with metastatic colorectal cancer, while additional alterations such as HER2 amplification and *NTRK* fusions are evaluated in selected clinical settings depending on testing availability and therapeutic eligibility [2,3]. The increasing implementation of precision oncology strategies has significantly modified the therapeutic landscape of colorectal liver metastasis (CRLM), allowing biomarker-driven treatment selection and improved patient stratification [2,3] (Table 4).

*KRAS* and *NRAS* mutations represent the most common actionable alterations in metastatic colorectal cancer and are identified in approximately 45–55% of metastatic cases overall [10,11,12]. These mutations lead to constitutive activation of downstream MAPK signaling pathways, promoting tumor proliferation, angiogenesis, invasion, and metastatic dissemination independent of EGFR signaling [10,11,12]. Clinically, RAS mutations are strongly associated with resistance to anti-EGFR monoclonal antibodies such as cetuximab and panitumumab, making wild-type RAS status mandatory before initiation of anti-EGFR therapy [10,11,12]. Several pivotal studies demonstrated that patients with RAS-mutant tumors derive minimal or no survival benefit from EGFR-targeted strategies and may even experience detrimental outcomes when anti-EGFR therapy is combined with certain chemotherapy regimens [10,11,12]. In addition to predictive implications, *KRAS* mutations have also been associated with more aggressive liver-dominant metastatic behavior, increased recurrence after hepatic resection, and inferior overall survival [10,11,12].

*BRAF V600E* mutation defines a biologically distinct and highly aggressive molecular subgroup occurring in approximately 8–10% of metastatic colorectal cancers [13]. This alteration results in constitutive activation of the MAPK pathway and is associated with poor differentiation, right-sided primary tumors, extensive metastatic burden, rapid progression, and significantly reduced survival [13]. Historically, conventional chemotherapy alone produced poor outcomes in *BRAF*-mutated disease, with median overall survival often below 12 months in advanced metastatic settings [15]. More recently, targeted combinations incorporating *BRAF* inhibition together with EGFR blockade have demonstrated clinically meaningful survival improvements compared with historical standard approaches [13]. These developments established *BRAF V600E* not only as a prognostic biomarker but also as an important predictive factor guiding targeted therapeutic strategies in metastatic colorectal cancer [13].

MSI-H/dMMR tumors represent a distinct immunogenic subgroup characterized by defective DNA mismatch repair, accumulation of insertion–deletion mutations, and high tumor mutational burden [14,15,16]. The resulting neoantigen-rich phenotype enhances tumor immunogenicity and partly explains the remarkable sensitivity of these tumors to immune checkpoint inhibition [14,15,16]. MSI-H/dMMR tumors account for approximately 10–15% of colorectal cancers overall but are less common in metastatic disease [14,15,16]. Clinical trials evaluating pembrolizumab and nivolumab-based regimens demonstrated durable responses, prolonged progression-free survival, and superior activity compared with conventional chemotherapy in MSI-H metastatic colorectal cancer [14,15,16]. Consequently, routine MSI/dMMR testing is now considered mandatory in metastatic colorectal cancer because it directly influences first-line systemic treatment selection [2,3,14,15,16]. Nevertheless, immune response heterogeneity persists, particularly in patients with liver metastases, where the hepatic immune microenvironment may contribute to immune exclusion and reduced immunotherapy responsiveness [14,15,16].

HER2 amplification is less frequent in colorectal cancer, occurring in approximately 2–5% of metastatic cases, but represents an increasingly important therapeutically actionable alteration, particularly in *RAS* wild-type tumors after standard therapy failure [29,30,31,32]. HER2-positive colorectal cancers often exhibit resistance to anti-EGFR therapy and may demonstrate aggressive biological behavior [29,30,31,32]. However, several HER2-targeted approaches—including trastuzumab-based combinations, trastuzumab deruxtecan, tucatinib plus trastuzumab, and dual HER2 blockade strategies—have shown clinically meaningful activity in selected HER2-positive metastatic colorectal cancer cohorts [29,30,31,32]. These therapies produced objective responses and durable disease control even in heavily pretreated patients, supporting the integration of HER2 testing into expanded molecular profiling algorithms for metastatic disease [29,30,31,32].

*POLE* and *POLD1* mutations are rare but biologically relevant alterations involving DNA polymerase proofreading dysfunction, leading to ultra-mutated tumor phenotypes with exceptionally high mutational burdens [33,34]. Similar to MSI-H tumors, these hypermutated cancers may generate enhanced neoantigen expression and increased sensitivity to immune checkpoint inhibition, including in selected microsatellite-stable tumors [33,34]. Although current evidence remains limited due to the rarity of these mutations, emerging studies suggest that *POLE/POLD1* alterations may identify a unique subset of patients potentially benefiting from immunotherapy despite MSS status [33,34]. At present, however, their routine clinical implementation remains less established than MSI/dMMR testing and continues to evolve primarily within translational and precision oncology research frameworks [33,34].

PD-L1 expression is biologically relevant within the colorectal cancer immune microenvironment because it contributes to immune suppression through interaction with the PD-1 receptor on cytotoxic T lymphocytes [28]. PD-L1 may be expressed not only by tumor cells but also by stromal cells, macrophages, dendritic cells, and other immune populations within metastatic lesions [28]. Despite its mechanistic relevance, PD-L1 expression alone has not demonstrated consistent predictive value for immunotherapy response in colorectal cancer and is not currently recommended as a standalone biomarker for checkpoint inhibitor selection [16,17,18,28]. In clinical practice, MSI/dMMR status remains substantially more reliable and clinically actionable than PD-L1 expression for identifying patients likely to benefit from immune checkpoint blockade [14,15,16,28].

#### 3.6.1. cDNA Analysis as a Predictive Factor in Patients with Colorectal Cancer Liver Metastasis (CRLM)

In the case of colorectal cancer with liver metastasis (CRLM), cDNA analysis offers critical insights into the molecular alterations that drive metastatic progression [24,25,26,27,28]. This method allows for the identification of differential gene expression between primary tumors and metastatic lesions, thus revealing potential biomarkers for early detection and therapeutic targets for intervention [24,25,26,27,28]. Liver metastasis in CRC is a multifaceted process involving complex interactions between tumor cells and the liver microenvironment [7,18,24,25,26,27,28]. This process unfolds in several key stages: invasion, extravasation, colonization, and immune evasion [24,25,26,27,28]. cDNA-based studies have provided invaluable data regarding the gene expression profiles and signaling pathways that underpin each of these stages of metastasis [24,25,26,27,28] (Table 5).

#### 3.6.2. Invasion and Migration

The invasion and migration of tumor cells are essential steps for metastasis, with the epithelial–mesenchymal transition (EMT) being a critical event that enables tumor cells to acquire migratory and invasive properties [24,25,26,27,28]. cDNA analyses have identified several genes associated with EMT that are frequently upregulated in CRC liver metastasis, including *Twist1*, *Snail*, and *ZEB1*, all of which promote cell motility and invasiveness [24,25,26,27,28]. A notable study by employed RNA sequencing to compare the gene expression profiles of primary CRC tumors and corresponding liver metastases [24]. The findings indicated significant upregulation of EMT-associated genes, such as *Vimentin*, *N-cadherin*, and *MMP-9* (matrix metalloproteinase-9), in metastatic liver lesions [24]. These results suggest that the acquisition of a mesenchymal phenotype is crucial for the metastatic spread of CRC to the liver [24].

#### 3.6.3. Colonization and Vascular Invasion

Upon reaching the liver, tumor cells must adapt to the liver microenvironment and initiate the formation of secondary tumors [7,12,24,25,26,27,28]. This process is facilitated by interactions with liver endothelial cells and the extracellular matrix (ECM) [24,25,26,27,28]. Key to this process are angiogenesis, the formation of new blood vessels, and vascular invasion, both of which are essential for tumor survival and expansion within the liver [24,25,26,27,28]. In one study, cDNA profiling of liver metastases from CRC patients revealed overexpression of *VEGF-A* (vascular endothelial growth factor A) and *HIF-1α* (hypoxia-inducible factor 1-alpha), both of which are known to promote angiogenesis [25]. Additionally, genes such as *CXCR4* (C-X-C motif chemokine receptor 4) were found to be upregulated in metastatic cells, enhancing vascular invasion and promoting successful colonization of the liver [25].

#### 3.6.4. Immune Evasion

The liver is an immune-privileged organ, and for metastatic tumor cells to thrive in this environment, they must evade immune surveillance mechanisms [7,14,15,16,18,24,25,26,27,28]. cDNA-based analyses have significantly advanced our understanding of the immune evasion strategies employed by metastatic CRC cells, particularly in terms of resistance to T-cell-mediated destruction [24,25,26,27,28]. cDNA microarray analysis on liver metastatic CRC samples and identified PD-L1 (Programmed Death-Ligand 1) as one of the upregulated genes [26]. The expression of PD-L1 on tumor cells is known to inhibit the activation of CD8+ T cells, effectively suppressing the immune response and allowing tumor cells to evade immune detection [14,15,16,26]. This finding has important therapeutic implications, particularly in the development of immune checkpoint inhibitors, such as nivolumab, which are being explored as treatment options for CRC patients with liver metastasis [14,15,16,26].

Tumor heterogeneity refers to the presence of diverse subpopulations of tumor cells within a single tumor mass [24,25,26,27,28]. This variability contributes to the adaptability of cancer cells, enabling them to exploit different niches within the body, including the liver [24,25,26,27,28]. cDNA-based studies have shed light on the genetic diversity within both primary and metastatic CRC tumors, revealing insights into the clonal evolution of cancer cells [24,25,26,27,28].

#### 3.6.5. Metastatic Subclones and Evolution

Through cDNA profiling, researchers have identified distinct subclones within metastatic lesions that exhibit specific gene expression profiles related to metastasis, chemoresistance, and immune evasion [24,25,26,27,28]. These subclones arise as a result of clonal evolution during CRC progression, and their characterization is crucial for understanding treatment resistance and tumor progression [24,25,26,27,28]. One study employed single-cell RNA sequencing on liver metastatic CRC samples and identified multiple subpopulations with distinct gene expression patterns [27]. One subclone exhibited high expression of EMT markers, while another demonstrated overexpression of immune checkpoint genes such as PD-L1 [27]. These findings suggest that subclonal evolution plays a pivotal role in driving treatment resistance and tumor progression in liver metastases [27].

Chemoresistance remains a significant challenge in the treatment of metastatic CRC [24,25,26,27,28]. cDNA analyses have been utilized to investigate gene expression profiles associated with chemotherapy resistance, particularly in liver metastases [24,25,26,27,28]. These studies have led to the identification of specific genes that may serve as potential targets for overcoming drug resistance [24,25,26,27,28]. Li et al. (2020) conducted cDNA microarray analysis of 5-fluorouracil (5-FU)-resistant liver metastatic CRC cells and identified the overexpression of *ABCB1* (ATP-binding cassette subfamily B member 1) [28]. This gene encodes P-glycoprotein, a drug efflux pump responsible for reducing the intracellular concentration of chemotherapy agents, thus contributing to resistance [28]. These findings highlight the role of drug transporters in chemoresistance and suggest that targeting these transporters could improve the efficacy of chemotherapy in CRC patients with liver metastasis [28].

cDNA-based approaches have also been instrumental in identifying potential biomarkers for the early detection, diagnosis, and prognosis of liver metastases in CRC [24,25,26,27,28]. By profiling gene expression patterns in liver metastatic lesions and comparing them to primary tumors, researchers have been able to pinpoint genes that are uniquely upregulated in the metastatic environment, offering potential targets for early detection and personalized treatment [24,25,26,27,28]. Several promising biomarkers identified through cDNA profiling have shown potential for clinical application in CRC metastasis [24,25,26,27,28]. cDNA microarray profiling to examine liver metastases from CRC patients and identified *GPC3* (Glypican 3) as a potential biomarker for liver-specific metastasis [28]. *GPC3* was found to be overexpressed in liver metastases but not in primary CRC tumors, making it a promising target for diagnostic imaging and therapeutic intervention [28].

The hierarchical classification presented in Table 6 illustrates the progressive transition from traditional histopathological prognostic factors toward integrated molecular and immune-based prognostic frameworks in colorectal liver metastasis (CRLM) [2,3]. Established biomarkers, including *KRAS*, *NRAS*, and *BRAF* mutations, MSI/dMMR status, lymph node involvement, vascular invasion, and tumor differentiation, remain the backbone of prognostic stratification due to their strong evidence base and incorporation into international clinical guidelines [2,3,10,11,12,13,14,15,16]. These biomarkers continue to influence therapeutic decision-making, particularly regarding anti-EGFR therapy eligibility, targeted therapy selection, and immunotherapy administration [10,11,12,13,14,15,16].

Conventional histopathological parameters remain clinically relevant because they reflect tumor aggressiveness, metastatic potential, and recurrence risk [8,9]. In particular, lymph node positivity, poor differentiation, vascular invasion, and tumor budding are consistently associated with inferior survival outcomes and increased metastatic dissemination [8,9]. At the molecular level, RAS pathway alterations are among the most clinically important predictive biomarkers, as *KRAS* and *NRAS* mutations are associated with resistance to anti-EGFR monoclonal antibodies and more aggressive disease biology [10,11,12]. Similarly, *BRAF V600E* mutation defines a distinct subgroup characterized by poor prognosis and limited responsiveness to conventional chemotherapy [13]. MSI-H/dMMR tumors represent a biologically distinct subset with increased tumor mutational burden and enhanced responsiveness to immune checkpoint inhibition [14,15,16].

Emerging biomarkers such as histopathological growth patterns (HGP), HER2 amplification, Immunoscore assessment, and immune microenvironment signatures reflect the growing emphasis on biologically driven patient stratification [6,7,8,9,17,29,30,31,32]. Desmoplastic HGP has been associated with improved survival and enhanced immune infiltration compared with non-desmoplastic growth patterns [8]. Tumor budding has also emerged as a marker of epithelial–mesenchymal transition and metastatic potential, particularly when integrated with additional stromal and molecular characteristics [9]. HER2 amplification, although relatively uncommon, represents a clinically actionable biomarker in selected patients with metastatic colorectal cancer [29,30,31,32].

The increasing recognition of the tumor immune microenvironment further supports the development of integrated prognostic models [6,7,17]. Immune-related parameters, including tumor-infiltrating lymphocytes (TILs), macrophage polarization, and Immunoscore evaluation, have demonstrated prognostic relevance in CRLM [6,17]. High densities of CD3+ and CD8+ lymphocytes correlate with improved oncological outcomes, whereas immunosuppressive macrophage populations and stromal remodeling contribute to tumor progression and immune escape [6,7,24,25,26,27,28].

Inflammatory biomarkers such as neutrophil-to-lymphocyte ratio (NLR), platelet-to-lymphocyte ratio (PLR), C-reactive protein-to-albumin ratio (CAR), and systemic immune-inflammation index (SII) represent accessible and cost-effective prognostic tools [19,20,21,22,23]. Elevated inflammatory indices have been associated with poorer survival, increased recurrence risk, and aggressive disease behavior in CRLM [19,20,21,22,23]. However, heterogeneity in cut-off values and study design currently limits standardization and widespread clinical implementation [19,20,21,22,23].

Experimental biomarkers, including transcriptomic signatures, cDNA profiling, *POLE/POLD1* mutations, and macrophage-based immune profiling, highlight the expanding role of translational oncology in CRLM [24,25,26,27,28,29,30,31,32,33,34]. These biomarkers provide important insights into tumor heterogeneity, immune evasion, angiogenesis, and treatment resistance, although prospective validation remains necessary before routine clinical integration [24,25,26,27,28,29,30,31,32,33,34].

## 4. Discussion

The prognosis of colorectal cancer (CRC) and its propensity to metastasize, particularly to the liver, is influenced by several histopathological and molecular factors [2,3,6,7,8,9,10,11,12,13,14,15,16,17,18]. Lymph node involvement, tumor differentiation, vascular emboli, and genetic markers play essential roles in determining the disease course and informing treatment strategies [2,3,8,9,10,11,12,13,14,15,16]. This discussion will explore how these factors contribute to the aggressiveness of CRC and its metastatic potential [2,3].

Lymph node positivity is a critical prognostic factor in CRC, strongly correlating with increased risks of distant metastasis, particularly to the liver [8,9]. Tumors that spread to the lymph nodes show poorer survival outcomes and more aggressive biological behavior [8,9]. The association of lymph node metastasis with liver involvement highlights the aggressive nature of these cancers and supports the use of systemic treatment intensification in high-risk patients [8,9,19,20,21,22,23]. Studies evaluating metastatic CRC populations demonstrated that lymph node-positive disease is associated with increased recurrence risk and reduced overall survival following hepatic resection [8,9].

The degree of tumor differentiation provides vital information regarding tumor biology and metastatic potential [8,9]. Poorly differentiated tumors (G3) are associated with increased genomic instability, higher metastatic capacity, and inferior oncological outcomes compared with well-differentiated tumors (G1) [8,9]. These findings emphasize the importance of tumor differentiation in prognostic assessment and therapeutic planning, particularly in patients considered for aggressive multimodal treatment strategies [8,9].

Vascular emboli and perineural invasion are histopathological features strongly associated with hematogenous dissemination and metastatic progression [8,9]. The presence of tumor cells within vascular or neural structures reflects invasive tumor behavior and correlates with increased recurrence risk and reduced survival [8,9]. These factors further support the importance of early systemic treatment and careful postoperative surveillance in high-risk CRLM populations [8,9,19,20,21,22,23].

Right-sided colon cancers (RCCs) possess distinct molecular characteristics, including higher frequencies of MSI-H status, *BRAF* mutations, CpG island methylation, and immune-related alterations [10,11,12,13,14,15,16]. These biological differences contribute to the more aggressive phenotype frequently observed in RCC and may partly explain the reduced responsiveness to anti-EGFR therapies compared with left-sided tumors [10,11,12,13]. Several studies demonstrated that primary tumor sidedness represents an important prognostic and predictive factor in metastatic colorectal cancer [10,11,12,13].

The histopathological growth pattern of liver metastases also significantly influences survival outcomes [8]. Desmoplastic growth patterns (dHGP) are generally associated with improved immune infiltration, clearer tumor margins, and superior survival compared with replacement-type or non-desmoplastic growth patterns [8]. In contrast, infiltrative and vessel co-opting metastatic patterns are associated with more aggressive disease biology and poorer oncological outcomes [8].

Tumor budding has emerged as an independent marker of invasion, epithelial–mesenchymal transition, and metastatic dissemination [9]. High-grade tumor budding correlates with increased recurrence rates, inferior survival, and reduced responsiveness to neoadjuvant therapy [9]. These findings suggest that tumor budding may contribute additional prognostic information beyond conventional staging systems, particularly when integrated with molecular and stromal characteristics [8,9].

Immunohistochemical (IHC) markers have provided additional insight into metastatic progression and therapeutic vulnerability in CRC [6,14,15,16,17,29,30,31,32]. Biomarkers such as CDX2, p53, HER2, mismatch repair proteins, and immune cell markers contribute to the characterization of tumor lineage, molecular subtype, and immune contexture [14,15,16,29,30,31,32]. Loss of CDX2 expression and abnormal p53 expression have both been associated with aggressive tumor behavior and inferior survival [29,30,31,32]. HER2 amplification represents a therapeutically actionable alteration in selected metastatic CRC subgroups [29,30,31,32].

The immune microenvironment within liver metastases represents a major determinant of treatment response and survival [6,7,14,15,16,17]. An inflamed phenotype characterized by tumor-infiltrating lymphocytes (TILs) and increased CD3+/CD8+ immune-cell density is generally associated with improved oncological outcomes [6,17]. Conversely, immunosuppressive macrophage populations, stromal remodeling, and immune checkpoint activation contribute to immune escape and metastatic progression [7,18,24,25,26,27,28]. The hepatic microenvironment itself possesses intrinsic tolerogenic properties that may facilitate metastatic colonization and reduce responsiveness to immunotherapy in some settings [7,14,15,16,18].

Systemic inflammation also plays an important role in CRLM progression [19,20,21,22,23]. Biomarkers such as neutrophil-to-lymphocyte ratio (NLR), platelet-to-lymphocyte ratio (PLR), C-reactive protein-to-albumin ratio (CAR), and systemic immune-inflammation index (SII) have demonstrated prognostic relevance in multiple studies [19,20,21,22,23]. Elevated inflammatory indices reflect a pro-tumoral inflammatory state associated with immune suppression, angiogenesis, tumor-cell survival, and metastatic dissemination [19,20,21,22,23]. Although these biomarkers are inexpensive and widely available, heterogeneity in cut-off values and study design currently limits their standardization in clinical practice [19,20,21,22,23].

The molecular profiling of colorectal liver metastasis (CRLM) has substantially improved the understanding of metastatic biology and precision oncology strategies [2,3,10,11,12,13,14,15,16]. Mutations involving *KRAS*, *NRAS*, and *BRAF* represent some of the most clinically relevant molecular alterations because they influence prognosis, treatment resistance, and therapeutic selection [10,11,12,13]. *KRAS* mutations are associated with constitutive MAPK pathway activation and resistance to anti-EGFR therapies such as cetuximab and panitumumab [10,11,13]. Similarly, *NRAS* alterations contribute to aggressive tumor behavior and limited responsiveness to EGFR-targeted therapies [10,11,13].

The presence of *BRAF V600E* mutation identifies a particularly aggressive molecular subgroup associated with poor prognosis and inferior survival [13]. The introduction of targeted combinations involving *BRAF* and EGFR inhibition has improved outcomes in this subgroup compared with historical chemotherapy-based approaches [13].

Microsatellite instability (MSI-H/dMMR) represents another clinically important biomarker because these tumors exhibit high tumor mutational burden and increased responsiveness to immune checkpoint inhibition [14,15,16]. Clinical trials evaluating pembrolizumab and nivolumab demonstrated substantial activity in MSI-H metastatic colorectal cancer, supporting routine MSI/dMMR testing in advanced disease [14,15,16]. Nevertheless, immune exclusion within liver metastases may partially reduce immunotherapy efficacy in certain patients [7,14,15,16,18].

HER2 amplification represents an increasingly relevant therapeutic target in selected metastatic CRC populations [29,30,31,32]. HER2-targeted regimens, including trastuzumab-based combinations, trastuzumab deruxtecan, and tucatinib plus trastuzumab, demonstrated promising activity in HER2-positive metastatic colorectal cancer cohorts [29,30,31,32]. These findings further support the implementation of expanded molecular profiling strategies in metastatic disease [2,3,29,30,31,32].

Although less common, *POLE* and *POLD1* mutations are biologically important because they define ultra-mutated phenotypes potentially sensitive to immune checkpoint inhibition [33,34]. However, their role in routine clinical decision-making remains investigational, and further validation is required before widespread implementation [33,34].

**Figure 3:**
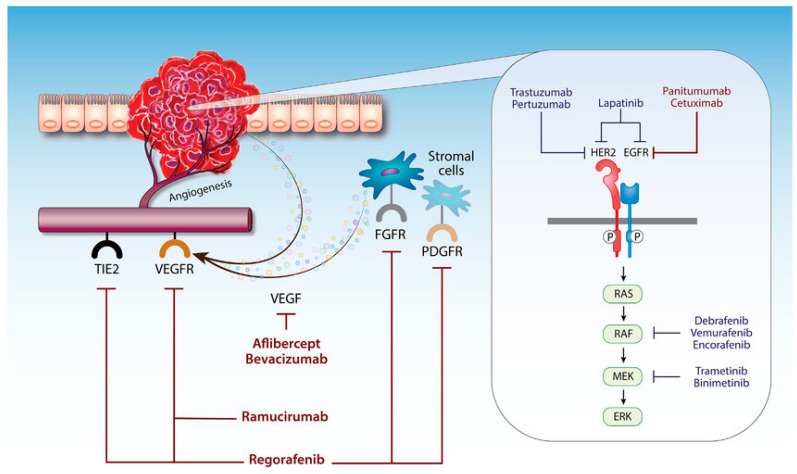
Biomarker-guided therapeutic algorithm in metastatic colorectal cancer. The figure summarizes sequential biomarker assessment, including RAS/RAF mutational status, MSI/dMMR testing, HER2 amplification, and selected rare actionable alterations to guide anti-EGFR therapy, *BRAF*-targeted treatment, HER2-directed therapy, and immune checkpoint inhibition. Histopathological and inflammatory risk factors are integrated into multidisciplinary therapeutic decision-making.

Treatment selection in metastatic colorectal cancer is increasingly guided by molecular biomarkers and precision oncology principles [2,3]. Current ESMO and NCCN guidelines recommend routine assessment of RAS mutations, *BRAF V600E*, MSI/dMMR status, and HER2 amplification in selected patients [2,3]. These biomarkers guide eligibility for anti-EGFR therapy, *BRAF*-targeted combinations, HER2-directed therapy, and immune checkpoint inhibition [2,3,10,11,12,13,14,15,16,29,30,31,32]. The integration of molecular biomarkers into therapeutic decision-making pathways is summarized in Figure 3.

Complementary DNA (cDNA) analysis has also expanded the understanding of metastatic progression in CRLM [24,25,26,27,28]. Transcriptomic profiling studies identified several genes involved in epithelial–mesenchymal transition, angiogenesis, vascular invasion, immune evasion, and chemoresistance, including *Twist1*, *Snail*, *ZEB1*, *VEGF-A*, *CXCR4*, and *ABCB1* [24,25,26,27,28]. These findings suggest that transcriptomic approaches may contribute to future biomarker discovery and therapeutic stratification [24,25,26,27,28].

The identification of biomarkers such as *GPC3* for liver-specific metastasis further highlights the potential of cDNA-based technologies in translational oncology research [24,25,26,27,28]. However, despite significant progress in molecular profiling and targeted therapies, several challenges remain. Tumor heterogeneity, immune exclusion, stromal remodeling, and acquired drug resistance continue to complicate treatment strategies in CRLM [7,18,24,25,26,27,28].

Overall, the integration of histopathological, molecular, inflammatory, and immune biomarkers represents a major step toward precision oncology in colorectal liver metastasis [2,3,6,7,8,9,10,11,12,13,14,15,16,17,18,19,20,21,22,23,24,25,26,27,28,29,30,31,32,33,34]. Continued translational and clinical research is necessary to refine biomarker-driven therapeutic strategies, improve patient stratification, and optimize long-term oncological outcomes in metastatic colorectal cancer [2,3,6,7,8,9,10,11,12,13,14,15,16,17,18,19,20,21,22,23,24,25,26,27,28,29,30,31,32,33,34].

## 5. Limitations of the Review

This review is limited by its narrative design, which does not allow quantitative synthesis or formal assessment of pooled effect sizes [2,3]. The included literature is heterogeneous with respect to study design, biomarker measurement, treatment era, systemic therapy exposure, surgical selection, and follow-up duration [2,3,4,5,19,20,21,22,23].

Several biomarkers discussed in this review, including inflammatory indices, HGP, tumor budding, immune-cell profiling, and transcriptomic signatures, are affected by inconsistent cut-off values or incomplete standardization [8,9,19,20,21,22,23,24,25,26,27,28]. Consequently, their interpretation should be cautious and context-dependent until larger prospective validation studies are available [8,9,19,20,21,22,23,24,25,26,27,28].

Some historical survival data were generated before routine use of contemporary molecular testing, targeted therapies, and immune checkpoint inhibitors [2,3,10,11,12,13,14,15,16]. Therefore, older outcome estimates may not fully reflect prognosis in the modern biomarker-driven treatment era [2,3,10,11,12,13,14,15,16,29,30,31,32].

## 6. Future Research Directions

Future CRLM research should prioritize integrated prognostic models that combine pathology, molecular alterations, inflammatory markers, immune contexture, and transcriptomic profiles [6,7,8,9,17,18,19,20,21,22,23,24,25,26,27,28,29]. Such models may better reflect disease biology than single biomarkers and may improve selection for liver resection, systemic therapy, targeted therapy, and immunotherapy [2,3,10,11,12,13,14,17,18,24,25,26,27,28,29,30,31,32].

Prospective multicenter studies are needed to standardize the assessment of HGP, tumor budding, inflammatory indices, and immune microenvironment signatures [8,9,19,20,21,22,23]. Spatial transcriptomics and single-cell sequencing may further clarify how tumor cells, stromal components, and immune populations interact during liver colonization and treatment resistance [24,25,26,27,28].

Clinical trials should increasingly incorporate biomarker-defined subgroups rather than treating CRLM as a biologically uniform disease [2,3,24,25,26,27,28,29]. This approach may improve the precision of therapeutic algorithms and support more individualized treatment pathways for patients with metastatic colorectal cancer [2,3,10,11,12,13,14,15,16,29,30,31,32,33,34].

## 7. Conclusions

CRLM progression is driven by a complex interplay of histopathological aggressiveness, molecular alterations, systemic inflammation, and immune microenvironment dynamics [2,3,4,5,6,7,8,9,17,18,19,20,21,22,23,24,25,26,27,28,29]. Classical prognostic factors such as lymph node involvement, tumor differentiation, vascular invasion, and tumor budding remain essential, but their clinical value is strengthened when interpreted alongside molecular and immune biomarkers [4,5,6,7,8,9,17,18].

Molecular profiling has transformed metastatic colorectal cancer management by identifying actionable alterations such as RAS/RAF mutations, MSI/dMMR status, HER2 amplification, and rare hypermutated phenotypes associated with POLE/POLD1 mutations [10,11,12,13,14,15,16,29,30,31,32,33,34]. Inflammatory biomarkers and transcriptomic signatures may further refine risk stratification, although additional validation is required before broad clinical implementation [19,20,21,22,23,24,25,26,27,28].

A biomarker-driven approach integrating histopathology, molecular oncology, systemic inflammation, and immune contexture offers the most coherent framework for future CRLM management [2,3,4,5,6,7,8,9,17,18,19,20,21,22,23,24,25,26,27,28,29,30,31,32,33,34]. Such multidimensional stratification may improve treatment selection, identify high-risk patients, and support more personalized therapeutic strategies for colorectal cancer patients with liver metastases [2,3,10,11,12,13,14,15,16,24,25,26,27,28,29,30,31,32,33,34].

## Figures and Tables

**Table 2:** The predictive character of the growth pattern of CRLM.

Pattern	Biological Interpretation	Clinical Implication
Desmoplastic	Fibrotic rim and organized stromal reaction	Generally associated with better prognosis [8,18]
Pushing	Expansile tumor-liver interface	Intermediate biological behavior [8]
Replacement	Cancer cells replace hepatocyte plates and co-opt vessels	Associated with aggressive biology and poorer outcomes [8,17]
Mixed	Combination of distinct interfaces	Requires careful standardized reporting [8]

**Table 3:** Overview of the Importance of Inflammatory Parameters and Cut-off Values in Relation to the Prognosis of Patients with Colorectal Cancer Liver Metastasis (CRLM).

Inflammatory Marker	Cut-off Value	Prognostic Significance	Study Example
NLR (Neutrophil-to-Lymphocyte Ratio)	≥3.0	High NLR is associated with poor overall survival (OS) and disease-free survival (DFS) after liver resection.	[19]
	≥4.0	Elevated NLR predicts increased recurrence and decreased survival in CRLM patients undergoing liver resection.	[20]
PLR (Platelet-to-Lymphocyte Ratio)	≥150	Elevated PLR correlates with worse prognosis, higher recurrence rates, and poor survival in CRLM patients.	[21]
	≥200	High PLR is associated with worse outcomes in CRLM patients treated with chemotherapy and liver surgery.	[21]
CAR (C-Reactive Protein-to-Albumin Ratio) (CRP mg/L ÷ albumin g/L)	≥0.3	Higher CAR indicates poor prognosis, lower survival, and higher recurrence risk in CRLM patients undergoing liver resection.	[22]
	≥0.5	Elevated CAR predicts poor response to chemotherapy and reduced survival in CRLM patients.	[22]
SII (Systemic Immune-Inflammation Index) (platelets × neutrophils/lymphocytes)	≥1000	SII ≥ 1000 is significantly associated with poor survival, higher recurrence, and aggressive disease in CRLM patients.	[23]
	≥800	Elevated SII predicts poorer prognosis in CRLM patients undergoing liver resection and chemotherapy.	[23]

**Table 4:** Predictive value, clinical significance and prevalence of *KRAS*, *NRAS*, *BRAF* V600, MSI-H/dMMR, HER2+, POLE/POLD1 in CRLM.

Biomarker	Prevalence in CRLM	Clinical/Prognostic Significance (ESMO/NCCN-Aligned)	Predictive Value for Treatment Selection	Key Evidence
*KRAS* mutation	~40–50%	Negative prognostic biomarker; associated with inferior overall survival, increased risk of liver-dominant metastatic disease, and aggressive tumor biology.	Predictive of primary resistance to anti-EGFR monoclonal antibodies (cetuximab, panitumumab). Median OS ~12 months versus ~21 months in RAS wild-type disease.	[10]
*NRAS* mutation	~5–10%	Similar adverse prognostic impact to *KRAS*; associated with aggressive disease course and early metastatic spread.	Predictive of lack of benefit from anti-EGFR therapy; classified together with *KRAS* as RAS-mutant disease per guidelines.	[11,12]
*BRAF* V600E mutation	~8–10%	Strong negative prognostic biomarker; associated with poor survival (median OS 15–18 months), rapid progression, and frequent liver metastases.	Predictive of poor response to standard chemotherapy; benefit from *BRAF*-targeted combination regimens (*BRAF* ± MEK inhibitors with EGFR inhibition).	[13]
MSI-H/dMMR	~10–15%	Favorable prognostic biomarker in early disease; distinct immune-driven tumor biology.	Strong predictive biomarker for immune checkpoint inhibitors (anti–PD-1 therapy). Limited benefit from fluoropyrimidine monotherapy.	[14,15,16]
HER2 amplification (ERBB2)	~2–5%	Associated with aggressive phenotype and poor response to standard therapies; more common in RAS wild-type tumors.	Predictive of response to HER2-targeted therapies (trastuzumab-based combinations), especially after failure of standard lines.	[29,30,31,32,33]
POLE/POLD1 mutations	Rare	Ultra-mutated phenotype; associated with high tumor mutational burden and distinct molecular subgroup.	Emerging predictive biomarker for response to immune checkpoint inhibition, even in microsatellite-stable tumors.	[33,34]
PD-L1 expression	Variable	Not considered a standalone prognostic biomarker in CRC per guidelines.	Not required for immunotherapy selection in CRC; limited predictive value compared with MSI/dMMR status.	[28]

**Table 5:** Summary of Studies on Gene Expression Profiles in Colorectal Cancer Liver Metastasis (CRLM).

Study	Objective	Methodology	Key Findings
[24]	To compare gene expression between primary CRC and liver metastases	RNA sequencing (RNA-seq)	Genes involved in epithelial–mesenchymal transition (EMT), such as Vimentin, N-cadherin, and MMP-9, were significantly upregulated in liver metastases, indicating that EMT is critical for CRC metastasis to the liver.
[25]	To profile gene expression in liver metastases and identify angiogenesis pathways	cDNA profiling	VEGF-A and HIF-1α were overexpressed, promoting angiogenesis. Additionally, CXCR4 upregulation facilitated vascular invasion, aiding liver colonization in CRC metastasis.
[26]	To investigate immune evasion mechanisms in liver metastatic CRC	cDNA microarray analysis	PD-L1 was found to be upregulated in liver metastatic CRC, inhibiting CD8+ T cell activation and facilitating immune evasion, suggesting potential for immune checkpoint therapy.
[27]	To identify metastatic subpopulations and their gene expression profiles	Single-cell RNA sequencing	Distinct subclones in liver metastatic CRC exhibited unique gene expression, with one subclone showing high EMT marker expression and another overexpressing immune checkpoint genes like PD-L1, contributing to tumor progression.
[28]	To investigate gene expression profiles related to chemoresistance in liver metastases	cDNA microarray analysis	ABCB1 (P-glycoprotein) was overexpressed in 5-FU-resistant liver metastatic CRC, suggesting its role in chemotherapy resistance by effluxing chemotherapeutic agents, pointing to potential drug transporter-targeted therapies.
[28]	To identify biomarkers for liver-specific metastasis in CRC	cDNA microarray profiling	GPC3 (Glypican 3) was overexpressed in liver metastatic CRC but not in primary tumors, highlighting its potential as a diagnostic and therapeutic target for liver metastasis in CRC patients.

**Table 6:** Hierarchical classification of prognostic and predictive biomarkers in colorectal liver metastasis (CRLM).

Biomarker Category	Specific Factor	Evidence Tier	Strength of Prognostic Impact	Predictive Role in Therapy Selection	Clinical Implementation Status
ESTABLISHED FACTORS (Guideline-supported)	*KRAS*/*NRAS* mutation	Established	Strong negative prognostic value	Anti-EGFR resistance	Routine molecular testing
	*BRAF* V600E mutation	Established	Very poor survival outcomes	Targeted therapy combinations	Standard testing in metastatic CRC
	MSI-H/dMMR status	Established	Distinct immune phenotype	Immunotherapy eligibility	Mandatory biomarker
	Lymph node involvement	Established	Major predictor of metastasis and OS	Influences systemic therapy	Standard pathology reporting
	Tumor differentiation (G1–G3)	Established	Aggressiveness indicator	Indirect therapeutic implications	Routine clinical use
EMERGING FACTORS (Strong evidence, limited standardization)	Histopathological Growth Pattern (dHGP vs. non-dHGP)	Emerging High	Strong survival stratification	Potential surgical planning value	Limited to specialized centers
	Tumor budding	Emerging High	Independent predictor of metastasis	Possible neoadjuvant response marker	Not universally reported
	HER2 amplification	Emerging High	Aggressive phenotype	HER2-targeted therapy	Selected molecular panels
	Immunoscore/TIL density	Emerging High	Strong immune-related prognosis	Possible immunotherapy guidance	Limited clinical adoption
	NLR/PLR/CAR/SII	Emerging Moderate	Reflect systemic inflammatory burden	Risk stratification	Easily available but non-standardized
EXPERIMENTAL FACTORS (Translational research stage)	POLE/POLD1 mutations	Experimental	Hypermutated phenotype	Potential immunotherapy sensitivity	Research setting
	PD-L1 expression in CRLM	Experimental	Inconsistent prognostic role	Not required for therapy selection	Not guideline-supported
	cDNA/transcriptomic EMT signatures	Experimental	Associated with invasion and colonization	Future precision oncology target	Research only
	CD68−CD163+ macrophage profile	Experimental	Immunosuppressive niche marker	Potential immunomodulation target	Translational studies

## Data Availability

No new data were created or analyzed in this study. Data sharing is not applicable to this article.
